# RGB-IR Cross Input and Sub-Pixel Upsampling Network for Infrared Image Super-Resolution

**DOI:** 10.3390/s20010281

**Published:** 2020-01-03

**Authors:** Juan Du, Huixin Zhou, Kun Qian, Wei Tan, Zhe Zhang, Lin Gu, Yue Yu

**Affiliations:** 1 School of Physics and Optoelectronic Engineering, Xidian University, No. 2 South Taibai Road, Xi’an 710071, Chinatwtanwei1992@163.com (W.T.); zhangzhe0011@163.com (Z.Z.); 2 Research and Development of Infrared Detection Technology, China Aerospace Science and Technology Corporation, Shanghai 201109, China; jsdtqk@163.com; 3 Shanghai Aerospace Control Technology Institute, Shanghai 201109, China; 4 National Institute of Informatics, Tokyo 101-8430, Japan; ling@nii.ac.jp

**Keywords:** super-resolution, infrared image denoising, guided filter layer, sub-pixel convolution

## Abstract

Deep learning-based image super-resolution has shown significantly good performance in improving image quality. In this paper, the RGB-IR cross input and sub-pixel upsampling network is proposed to increase the spatial resolution of an Infrared (IR) image by combining it with a color image of higher spatial resolution obtained with a different imaging modality. Specifically, this is accomplished by fusion of the features map of two RGB-IR inputs in the reconstruction of an infrared image. To improve the accuracy of feature extraction, deconvolution is replaced by sub-pixel convolution to upsample image in the network. Then, the guided filter layer is introduced for image denoising of IR images, and it can preserve the image detail. In addition, the experimental dataset, which is collected by us, contains large numbers of RGB images and corresponding IR images with the same scene. Experimental results on our dataset and other datasets demonstrate that the method is superior to existing methods in accuracy and visual improvement.

## 1. Introduction

Image super-resolution (SR) has become a hot research topic to improve the image resolution by means of software algorithm. The key is to obtain an estimation of a High Resolution (HR) image from the Low Resolution (LR) input. Usually, Infrared (IR) detectors can be used for night video monitoring, biomedicine, forest fire fighting and safe driving. However, IR images captured by infrared imaging devices always suffer from low resolution, low contrast and blur details. Therefore, it is more difficult to extract information of interesting objects from IR images than that of color images.

Traditional research on image super-resolution mainly focuses on two aspects. One is trying to find the mapping relationship between HR and LR images with respect to pixels or local patches, which always involves Neighbor Embedding (NE) and Anchored Neighborhood Regression (ANR). Hong Chang introduced Neighbor Embedding [1], which can be reconstructed by its neighbors in the feature space and not highly depend on sample. Howerver, the number of fixed neighborhoods would cause overfitting or under-fitting problems. Radu Timofte, et al. proposed Anchored Neighborhood Regression [2,3] which takes the dictionary atom as the neighborhood to reduce the operational complexity and running time, but it loses flexibility. The other kind of method is sparse coding, which aims to learn the similarity between LR and HR patches via large datasets. Jianchao Yang presented the sparse coding algorithm  [4,5] via patch-based sparse coding and two dictionaries to achieve the image super resolution, which has great improvement in SR (super-resolution) quality. However, the dictionary is not complete and the image edge quality is not high. In summary, the traditional algorithm has limited SR effect on image super-resolution, and its speed is not fast.

Recently, end-to-end learning methods were successfully applied into the field of SR, object detection, obstacle recognition [6]. Dong et al. firstly combined the Convolutional Neural Network (CNN) method with SR, and proposed the Super Resolution Convolution Neural Network (SRCNN [7]) algorithm and Fast SRCNN (FSRCNN [8]), with better performance than traditional algorithms. J. Kim et. al proposed the Very Deep Convolutional Networks (VDSR [9]), which has 20 convolution layers to further exploit context information. To avoid the overfitting of deep networks, J. Kim et al. introduced the Deeply-Recursive Convolutional Network (DRCN [10]) with residual structure and skip-connections. In the last two years, many modules were dated to solve low resolution problems. Jiayi Ma [11] presented a dense discriminative network that is composed of several aggregation modules (AM) and aggregate features progressively in an efficient way. Still, the above algorithm deep learning network can only process a single data source, and can only use the characteristics of a single sensor. There are certain limitations to enhancing details. There are also some limitations can only handle a single sensor image.

Meanwhile, various approaches via multi-source data (infrared image and color image) were proposed to increase the image high-frequency information as much as possible [12]. To solve the problems of lighting, T. Y. Han [13] proposed a method which fuses multiple LR images taken at different camera positions to synthesize a HR image. C. W. Tseng [14] proposed a network in which infrared images and color images are concatenated as input to construct a high-resolution IR image. However, as single-input networks, these algorithms ignore the high-frequency information of visible images captured by visible detectors. In addition, most networks have not got trained network models that take paired infrared and visible images. To solve this problem, we propose a network to mine information of visible image for high-resolution images reconstruction. The network has multi-modal input of infrared and visible light images to make full use of the feature information of multiple sensors.

Infrared detectors can clearly display target images at night or partially occluded. In the limitation to the hardware system of Infrared detectors, the infrared images have a few problems: low resolution, blurring, and random noise. Apart from training convolutional networks with multi-sensors images as input, the key to achieving infrared image super-resolution is the removal of infrared noise. In this paper, we applied guided filter [15] to suppress the noise in IR images.

The structure of our RGB-IR cross input and sub-pixel up-sampling network is shown in Section 3. The main contributions are listed as follows: (1) Paired IR and RGB images are used as inputs, unlike most existing works which only take single-sensor images. (2) Sub-pixel convolution is applied to extract multi-channel features from RGB images, which is used to enhance the IR image. (3) Guided filter layer is adopted to reduce the influence of noise in IR images, which makes the IR image reconstruction effective. (4) Dataset of IR-COLOR2000 is captured by ourselves for training ideal network.

The specific operation is as follows. First, a guided filter layer is introduced to suppress the noise in IR images. Then, the RGB image is convoluted by sub-pixel convolution filter to obtain feature images, and the HR image is represented by the sum of the RGB image feature and IR upsampling image feature. In addition, we take our dataset, called IR-COLOR2000, as the dataset for training the ideal network. Finally, experimental results show that the proposed algorithm is superior to several comparison algorithms.

The remainder is organized as follows. Section 2 mainly contains the convolutional neural network for image SR, the existing datasets for super SR domain, and infrared image denosing algorithm. Section 3 presents the RGB-IR cross input and sub-pixel upsampling model, the importance of guided filter layer and sub-pixel convolution model. Section 4 follows the algorithm simulation for IR image and RGB image, respectively. Finally, conclusions are drawn in Section 5.

## 2. Related Works

### 2.1. CNN-Based SR

CNN-based works for SR algorithms basically focus on the single modal image (color image or infrared image). The Super-Resolution Convolution Neural Network (SRCNN) demonstrates that the three-layer network extracted the convolutional feature to reconstruct image, which runs at high speed but is very sensitive to noise. To extract deeper image features and avoid gradient attenuation, Kim proposed a Very Deep Convolutional Networks (VDSR) method, which learned only the high-frequency residuals between the input and the output. In addition, it reduced the training time of learning a large number of low-frequency similar parts. Moreover, the Laplacian Pyramid Super-Resolution Network (LapSRN [16]) can get the intermediate feature image and predict the next HR image by adding feature images. For the reason that the reconstruction HR image is gradually enlarged in the scale of 2, the algorithm running speed is relatively fast. However, these algorithms ignore the features of infrared images and have not yet got trained models from multimodal features of infrared and color  images.

The structure of the SRCNN, FSRCNN, VDSR, DRCN, and LapSRN networks are shown in Table 1. CNN-based super-resolution methods have many forms of network structure in Table 1, from the simplest 3-layer network to a 24-layer residual network. Among them, they are divided into direct reconstruction and progressive reconstruction according to the number of upsampled images. Methods with direct reconstruction perform pre-upsampling from the LR to HR patches, and progressive reconstruction is multiple post upsampling steps. The input of the network is LR or an interpolated image (LR + bicubic). Depth is the number of convolutional layers passed from input to 4x output. In addition, the loss function uses L2 loss function or other loss functions.

Compared with other network structures, LapSRN has the following advantages: progressive image reconstruction to reduce network parameters, residual structure to deepen the network, and use of the Charbonnier loss function suitable for the pyramid structure.

### 2.2. Laplacian Pyramid Network

**Figure 1:**
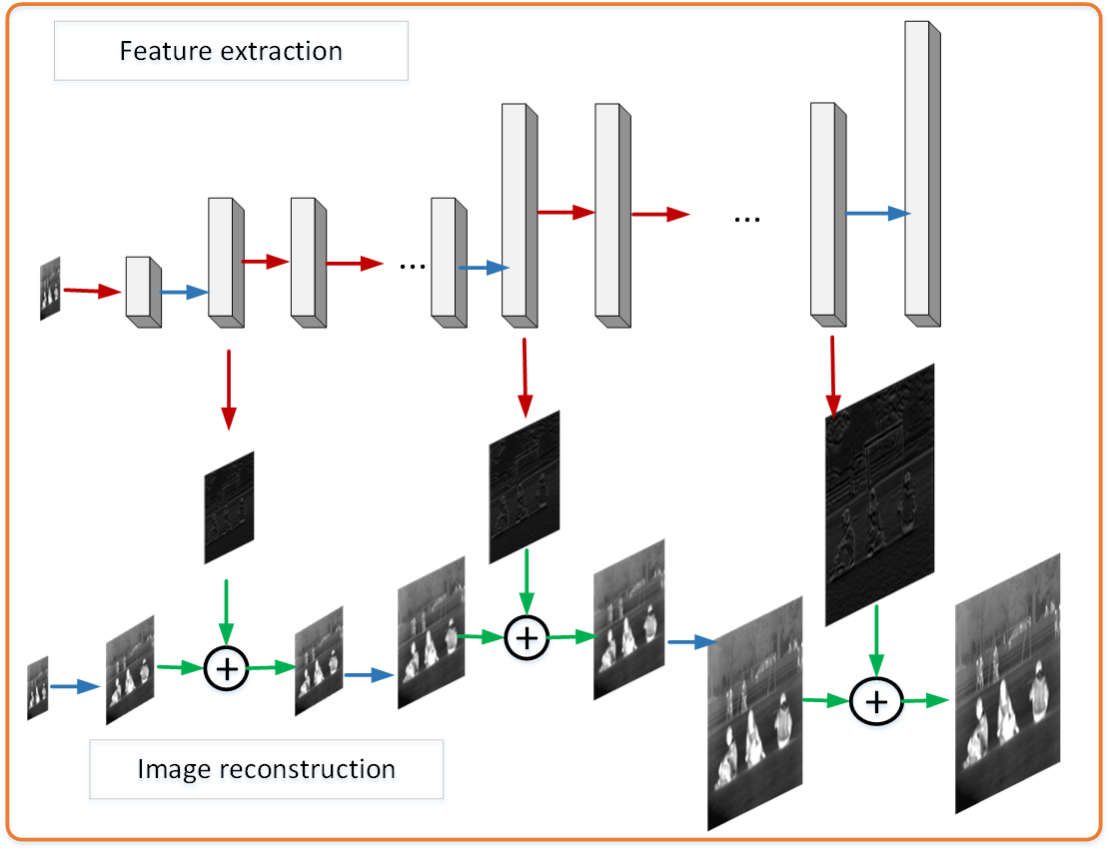
LapSRN Network Architecture

The mainstream framework of image SR is to study the end-to-end network over one type of image. In this section, a deep Laplacian pyramid network (LapSRN) [16] is introduced for SR. The LapSRN contains two sub-networks of feature extraction and image reconstruction as Figure 1. We extracted feature images in feature extraction branch, then sent the feature into upsampling images to get residual result in image reconstruction branch for high resolution. Furthermore, the network output HR image is obtained from RGB image features and upscaling images at each level. In other words, the network uses multiple pyramid layers, and each layer shares the extracted features with the upsampling image in progressive reconstruction at scale 2. After training on three pyramid layers network, the output we obtained is 8 times the input.

**Loss function**. The loss function is the same Charbonnier penalty function as LapSRN. To represent the difference between HR image and the SR image, we assumed that the residual image of the *s*-level pyramid is $r_{s}$, the image after the up-sampling is $x_{s}$, and the corresponding HR image is $y_{s}=r_{s}+x_{s}$. The corresponding pyramid level $Y_{s}$ is generated from downsampling the HR and the bicubic interpolation and loss function is

(1) $$L_{oss}\left(Y,y;\theta \right)=\frac{1}{N}\sum_{i=1}^{i=N}\sum_{S=1}^{S=L}\delta \left(Y_{s}-y_{s}\right)$$

(2) $$L_{oss}\left(Y,y;\theta \right)=\frac{1}{N}\sum_{i=1}^{i=N}\sum_{S=1}^{S=L}\delta \left(\left(Y-x_{s}\right)-r_{s}\right)$$

where $\delta \left(x\right)=\sqrt{X^{2}+\varepsilon^{2}}$ is explained δ() in Equation (2); X represents $\left(Y-x_{s}\right)-r_{s}$; ε is penalty, and the value is very small. *L* is the number of pyramid layer (*L* = 1, 2, 3); *i* denotes the pixels in image, *N* is number of pixels in image.

### 2.3. SR Datasets

Image super-resolution datasets are common datasets in the field of image processing. The most widely used datasets are ImageNet [17], Urban 100 [18], Greneral-100 [8], Set5, Set14, Manage109 [19], BSD300 and BSD500 [20]. The test data that is often used in image processing are Set5, Set14, Manage109, and so on. Databases of infrared and visible images [21,22,23] were applied in different computer vision tasks such as image enhancement, pedestrian detection, and region segmentation. However, most datasets do not consider IR images of high resolution. Therefore, we devote ourselves to capture pairs of IR and color images to train the deep network efficiently.

**Dataset of IR-COLOR2000**. The datasets contain both day and night images, which were generally captured in the outdoor (gardens, playgrounds, and buildings). The human in the night is salient in IR images, indicating that IR is sensitive to thermal radiation. Information about outdoor images is more sufficient than that of indoor images.

**Figure 2:**
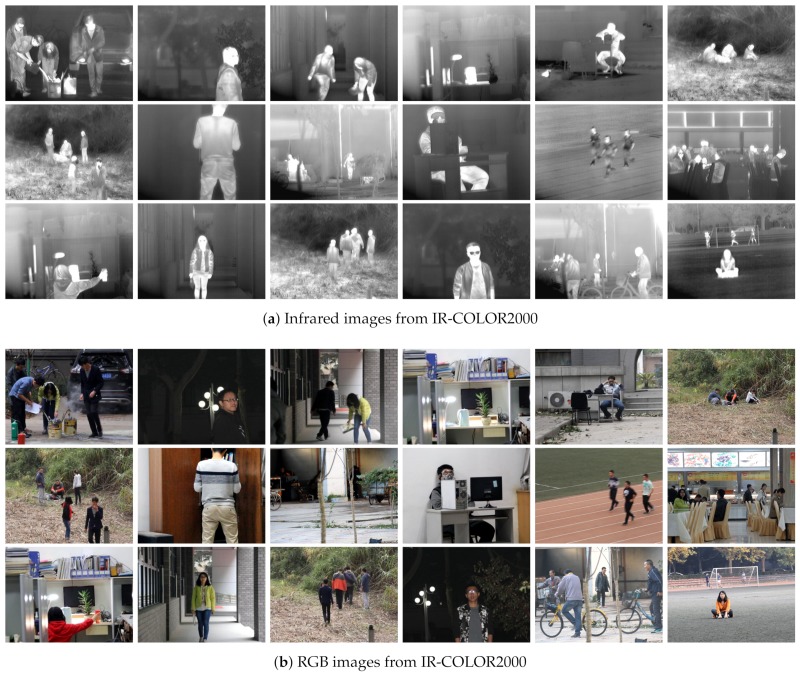
Dataset Section 2.3 of IR-COLOR2000( the scene in outdoor and indoor ), (**a**) Infrared images from IR-COLOR2000, (**b**) RGB images from IR-COLOR2000.

To effectively train the deep network, the database should contain large numbers or different types of images. In this paper, datasets that include IR and RGB images are obtained via two independent cameras, an uncooled long-wave detector IRT102, and Canon 60D. Besides, each pair of RGB and IR image faces the same environment. The data contains images of day and night, usually captured outdoors (gardens, playgrounds, and buildings). For the same scene image, the target size and the resolution of infrared images use the local self-similar-based image registration algorithm [24]. The database (named IR-COLOR2000) captured here consists of thousands of RGB and IR images with different illumination or scene. In particular, several pairs of images are shown in Figure 2.

### 2.4. IR Image Denoising

In the process of IR image SR, the image has low contrast and fuzzy edge. In particular, there are types of denoising algorithms, including variational, Total Variational (TV [25]), Partial Differential Equation (PDE [26,27]), Block-Matching, and the 3D filtering (BM3D [28,29]) method. TV is used to reduce the degradation of flat areas of the image, but it has faults of complex calculations and slow convergence. The purpose of PDE is to deal with fuzzy edges and edge position movement. PDE has a good effect on eliminating noise close to the Gaussian distribution, but it is not ideal for impulsive noise. BM3D is a noise reduction method that improves the sparse representation of images in the transform domain but the BM3D algorithm to perform random noise is not good.

Although the quality of IR images is improved, there is also much noise that needs to be reduced via images priors, such as smoothness and self-similarity. So the appropriate method for IR image denoising needs to be explored in the IR image super resolution procession without priors, such as [30]. Subsequently, the curvature filter [31] and guided filter methods [15,32], which can obtain well edge-preserving smoothing and less noise infrared images, are adopted for denoising. With a color image used as the guidance, the IR image can be well reconstructed, and the depth image edge can be preserved completely [14]. At the same time, we found that the network-based guided filtering [33] method can be trained to achieve an end-to-end network for image enhancement.

## 3. The Proposed Algorithm

**Figure 3:**
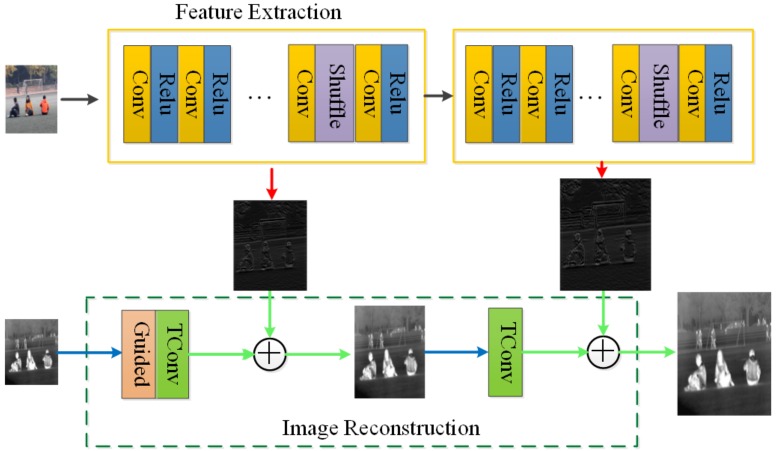
RGB-IR cross input and sub-pixel upsampling Network architecture. (the red arrow represents feature extraction, the green arrow represents the addition of the extracted feature and the upsampled feature, and the blue arrow represents the upsampling operation.).

In this section, based on LapSRN, this paper improves the LapSRN in three aspects for the characteristics of infrared images. we describe the proposed RGB-IR cross input and sub-pixel upsampling network. In the proposed network (Figure 3), pairs of IR and RGB images are used as input together, sub-pixel convolution is applied to optimize feature extraction network and a guided filter layer is adopted to reduce the influence of noise in IR images.

Several defects in IR image reconstruction, such as background noise, low contrast, and blur, result in bad performance. Therefore, an improved LapSRN based on IR-RGB images is presented to deal with IR image SR. RGB-IR cross input and sub-pixel upsampling network is also made up of RGB image feature extraction and IR image reconstruction. Sub-pixel convolution excels deconvolution in image upsampling to optimize feature extraction network especially. Moreover, the guided filter layer plays an important role in infrared image denoising and edges preserving.

### 3.1. RGB-IR Cross Input and Sub-Pixel Upsampling Network

Not to increase the depth of the network, the multi-modal image is used as input in our network for more details. That is, features of the visible image are add in infrared image reconstruction.

It is superior to other networks in that the proposed model includes sub-pixel layer for upsampling and a guided filtering layer for infrared image denoising. As Figure 3 shows, the features from RGB image is extracted by feature extraction group which concatenates the feature from “convolution” → “Relu” → “convolution” → “Relu” →⋯→ “sub-pixel convolution” → “convolution”. Then, the features from RGB images are added to the reconstruction of infrared images that go through “guided filtering” and “Deconvolution” to up-sampling processing. In this way, each image feature and infrared feature combination will enlarge the image scale by two times. Multi-scales $2^{N}$ image super-resolution needs to pass the feature to the next level for *N* feature extraction, and the infrared feature upsampling process, finally completes the reconstruction convolution. The detail information such as the kernel functions, depths and I/O dimensions of all “convs” in “Feature Extraction” and “Image Reconstruction” branches are shown in Table 2.

**Figure 4:**
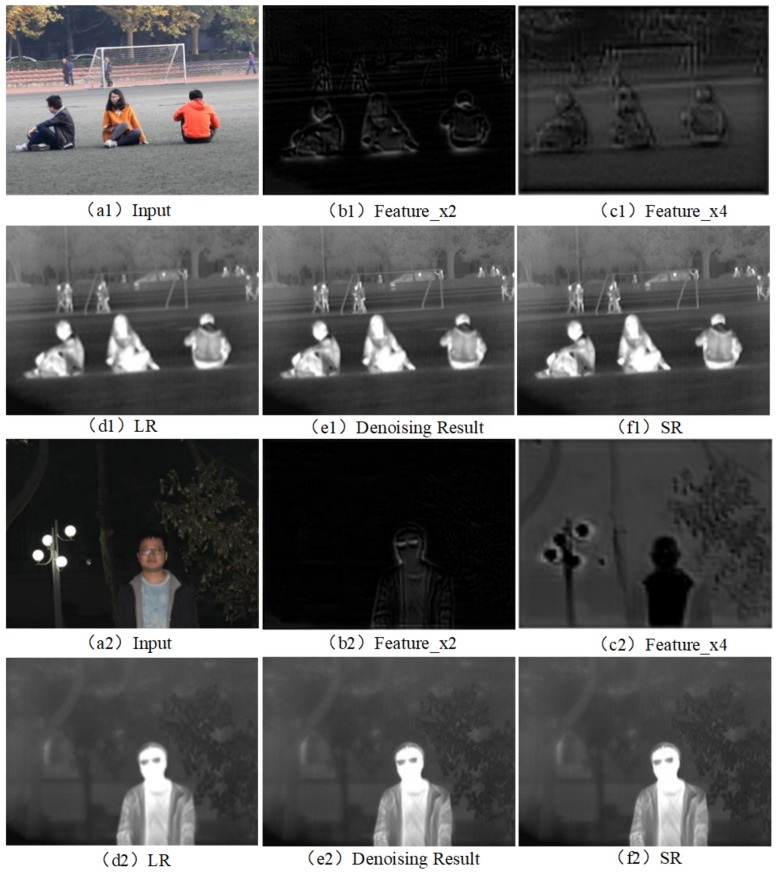
The input, output and intermediate layer image of RGB-IR cross input and sub-pixel upsampling network at ×4 scale. (**a**) input RGB image, (**b**) feature image ×2, (**c**) feature image ×4, (**d**) input IR image, (**e**) denoising result, and (**f**) SR image.

Network-intermediate layer images include feature image ×2, feature image ×4, and denoising results. Two sets of images are listed in Figure 4 which are the images of the middle layer in the proposed network at ×4 scale.

Residuals output of different scales and corresponding scale reconstruction results are obtained through cascade learning. The branch of image feature extraction takes RGB image as input to extract detailed features for the reconstruction of infrared image, and gradually samples and adds feature images of the same scale in the process of image reconstruction.

The feature extraction network extracts the visible image features, and upsamples the feature image through the sub-pixel convolution layer, and upsamples infrared image via the transposed convolution in the infrared image reconstruction, and the output image size is twice than the input image. When the super-resolution reconstruction scale is ×2, ×4, and ×8, the sub-pixel upsampling network feature extraction and the infrared image reconstruction upsampling operation are repeated m times (m = 1, 2, 3 ). The upsampling layer extracts image feature via a sub-pixel convolution layer. In each sub-pixel convolution part, the upsampled feature image is twice the size of its former feature image. It is worth noting that the input in the upsampling layer is IR images, and the RGB image is another input in the part of the feature extraction, which gives more detail information compared to handle SR.

### 3.2. Sub-Pixel Convolution

Taking into account that direct interpolation of deconvolution upsampling causes image blur, we have to use progressive upsampling for network upsampling and use subpixel convolution instead of deconvolution to reduce the loss of detail in the upsampling process. Sub-pixel convolution combines pixel values of multiple channels into one feature map, thus adding features in the network and changing the feature image size.

**Figure 5:**
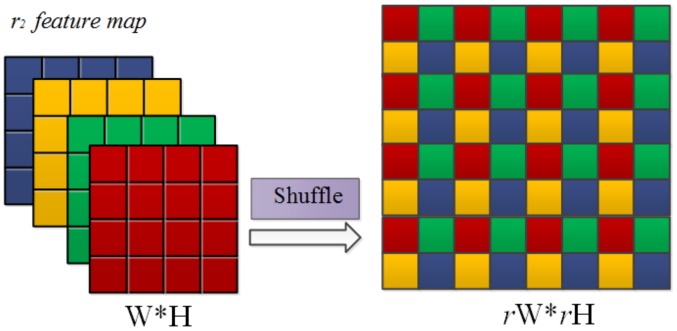
Sub-pixel convolution (*r* = 2).

The sub-pixel layer plays a role as upsampling achieved by sub-pixel convolution in the extraction network. In contrast with upsampling, sub-pixel convolution combines feature images from multiple channels into one image. Pixel values in feature images are multiple channels value at the same position. The sub-pixel convolution layer can upscale the final LR feature maps into the HR output in the paper [34]. The feature map $I^{F}$ is the input of sub-pixel upsampling layer; $f^{L}\left(I^{F}\right)$ is the output of sub-pixel upsampling layer; $W_{L}$ and $b_{L}$ is sub-pixel upsampling layer parameters. The implementation of sub-pixel convolution can be expressed as where ϕ( ) is sub-pixel convolution operation, called shuffle. That operation changes the feature shape from $W\times H\times r^{2}$ tensor to rW×rH. The effects of sub-pixel convolution operation are shown in Figure 5. Sub-pixel convolution combines a single pixel on a multi-channel feature into a single pixel on a feature. Figure 5 shows the operation of sub-pixel convolution intuitively. To upsample the feature map of *r* times size, we need to generate $r^{2}$ feature maps of the same size. The operation of sub-pixel convolution is to assemble $r^{2}$ same size feature maps into a larger *r* times map.

(3) $$f^{L}\left(I^{F}\right)=\phi \left(W_{L}*f^{\left(L-1\right)}\left(I^{F}\right)+b_{L}\right)$$

**Figure 6:**
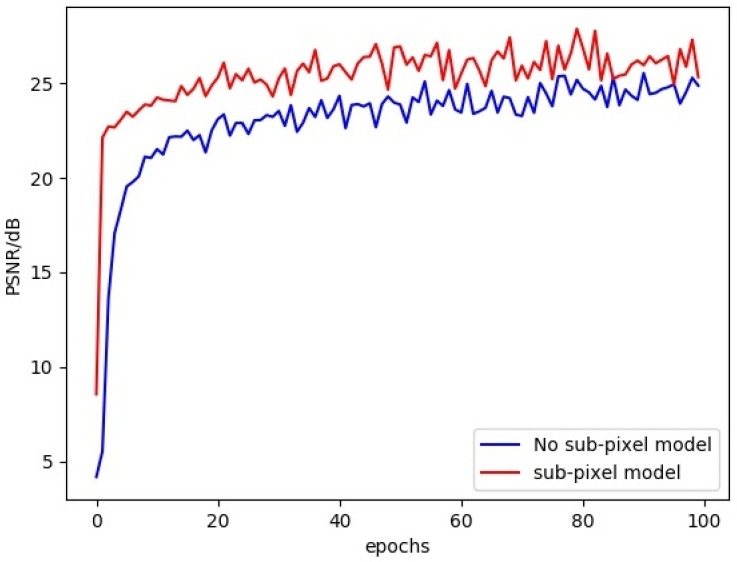
Performance of sub-pixel model in feature extraction. (blue curve represents no sub-pixel model, red curve represents sub-pixel model)

For the sake of improving and optimizing the network, sub-pixel convolution is used instead of deconvolution for image upsampling. Sub-pixel convolution avoids the danger of large numbers of zeros in general deconvolution. Besides, As can be seen from the comparison training in Figure 6, the sub-pixel convolution module has always had an absolute advantage over the simple upsampling in training 100 epochs on the dataset of IR-COLOR2000 for ×4 SR.

To prove the benefits of sub-pixel convolution, comparative experiments were conducted between networks with transposed convolutions and networks with sub-pixel convolution to improve network performance and rich feature details. As can be seen from the comparison training in Figure 6, the sub-pixel convolution module has always had an absolute advantage over the simple upsampling in training 100 epochs. The sub-pixel upsampling network in the feature extraction model makes full use of sub-pixel convolution. The experiment result shows that sub-pixel convolution is used for image feature extraction, which greatly improves the signal-to-noise ratio (PSNR) of the image.

### 3.3. Guided Filter Layer

Apart from training convolutional networks for more detail infrared image, the key to achieve infrared image super-resolution is the removal of infrared noise. It is necessary to enhance the infrared image and remove noise for uncooled long-wave infrared images containing noise. A denoising method is needed to eliminate noise and enhance the image in convolutional networks. Guided filtering has edge-preserving and denoising. It is through the network to train the guided filter parameters for image denoising enhancement.

The influence of noise can be reduced by the guided filter method. Furthermore, an RGB image is used as the guidance to complete IR image SR. The guided filter layer of this article is identical to the previous works in principle. The guided images we use are different. Generally, the input image is used as a guided image to maintain the edge. In this paper, the corresponding RGB image is used as a guided image to reduce the noise of the infrared image.

The guided filter process is linear translation-variant, and the output *q* is obtained by the guided filtering method of the input and the guidance *I*.

(4) $$q_{i}=a_{k}I_{i}+b_{k}$$

where, $a_{k}$ and $b_{k}$ are the linear coefficients with restriction of the window $w_{k}$; *k* is the center point of $w_{k}$ window. In the filtering calculation, *i* represents the pixels of the window $w_{k}$.

To seek a solution of minimizing the difference between output image *q* and input image *p*, it can also maintain the linear model Equation (4). The Equation (5) is the cost function in the window $w_{k}$. ϵ is a penalizing parameter.

(5) $$E\left(a_{k},b_{k}\right)=\sum_{i\in w_{k}}\left({\left(a_{k}I_{i}+b_{k}-p_{i}\right)}^{2}+\epsilon a_{k}^{2}\right)$$

(6) $$a_{k}=\frac{\frac{1}{\left|w\right|}\sum_{i\in w_{k}}I_{i}p_{i}-\mu_{k}\bar{p_{k}}}{\sigma_{k}^{2}+\epsilon }$$

(7) $$b_{k}=\bar{p_{k}}-a_{k}\mu_{k}$$

Here, $\mu_{k}$ and $\sigma_{k}^{2}$ are respectively represented as the mean and variance of *I* in $w_{k}$, $\left|w\right|$ is the number of pixels in $w_{k}$ and $\frac{1}{\left|w\right|}\sum_{i\in w_{k}}p_{i}$ is the mean of *p*.

The flow chart and step of the presented algorithm are shown in Algorithm 1 and Figure 3, respectively.
**Algorithm 1** Guided Filtering Layer for image processing **Input:** low-resolution IR image $I_{l}$           low-resolution RGB image $G_{l}$           Radius r and Regularization term ϵ **Output:** Guided filtered image as output $O_{h}$1. $G_{l}$ mean matrix calculation: $\bar{G_{l}}=f_{\mu }\left(G_{l},r\right)$2. $I_{l}$ mean matrix calculation: $\bar{I_{l}}=f_{\mu }\left(I_{l},r\right)$3. $G_{l}$ Correlation calculation: $\bar{G_{l}^{2}}=f_{\mu }\left(G_{l}*G_{l},r\right)$4. $G_{l}$, $I_{l}$ Correlation calculation: $\bar{G_{l}I_{l}}=f_{\mu }\left(G_{l}*I_{l},r\right)$5. $G_{l}$ Variance calculation: $\sum_{G_{l}}=\bar{G_{l}^{2}}-\bar{G_{l}}*\bar{G_{l}}$6. $G_{l}$, $I_{l}$ Covariance calculation: $\sum_{G_{l}I_{l}}=\bar{G_{l}I_{l}}-\bar{G_{l}}*\bar{G_{l}}$Calculate guided filtering parameters $a_{k}$ and $b_{k}$ according to Equations. (6-7):7. $a_{k}=\frac{\sum_{G_{l}I_{l}}}{\sum G_{l}+\epsilon }$8. $b_{k}=\bar{I_{l}}-A_{l}*\bar{G_{l}}$9. $O_{h}=a_{k}*G_{l}+b_{k}$/* $f_{\mu }\left(\right)$ is a mean filter

**Figure 7:**
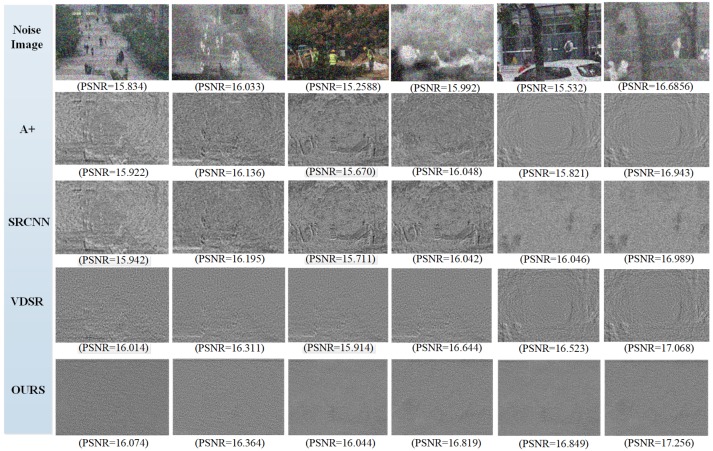
Difference image, which is A+, SRCNN, VDSR, ours SR result subtracting the input noise image. (PSNR values for noise image SR results.)

To eliminate image noise the guided filter layer was trained in the RGB-IR cross input and sub-pixel upsampling model, then compare the SR effect on the synthetic data, such as images added salt & pepper noise. The noise image as input is illustrated in Figure 7, to perform image super resolution simulation and check whether the model is sensitive to noise. In Figure 7, the denoising ability of algorithms was analyzed and judged by the difference image between the noise image and the SR result. The less information contained in the difference image, the better the image SR result and the denoising effect. In Figure 7, it can be clearly seen that the proposed method is better than the comparison algorithm. It can be seen from Figure 7 that the proposed method has a good effect of removing noise. However, it can be seen from the value of PSNR that the salt & pepper noise has a bad influence on the image SNR.

It is observed that four noise images referring to the A+ method and the SRCNN method still contain much noise and lose edge or texture information. However, both the VDSR method and the proposed method do well to reduce image noise. For further comparison, the proposed method is superior to the VDSR algorithm in a subjective visual sense. In summary, the guided filter can improve image quality and reduce IR noise effectively.

### 3.4. The Loss Function of Multi-scale

The network uses 2×, 4×, and 8× samples to train multi-scale super-resolution models. It is necessary to construct network structures at different scales (2, 4, 8) and reduce the differences between SR images and HR images at different scales.

(8) $$\sum_{scale=2,4,8}L_{scale}\left(Y,y,\theta \right)=L_{oss}\left(Y,y,\theta \right)$$

We note that the $L_{oss}\left(Y,y,\theta \right)$ is Charbonnier loss, and the scale in loss is upsampling scales for SR.

## 4. Experiment Results

### 4.1. Training & Testing

In this section, all experiments are implemented in Matlab 2016a on a PC with GPU Titan V, 12 GB RAM in ubuntu 16.04 system.

The training datasets (IR-COLOR2000), which contain 2000 pairs of images, are captured by ourselves. The images in the datasets main contain the infrared images and corresponding RGB images. Especially, we crop each input image into patches with a size of 128 × 128. The samples were generated by the operators of flipping horizontally and flipping vertically or rotating 90${}^{\circ }$, 180${}^{\circ }$, 270${}^{\circ }$. After the training is completed, the pre-trained model will be able to make 2×, 4× SR respectively. When testing, just select the model and enter the image to be tested.

We simultaneously conduct extensive evaluations of existing super-resolution models as baselines on our IR-COLOR2000 datasets. Then, the performance of several deep models, including the proposed RGB-IR cross input and sub-pixel upsampling network are evaluated. Three sets of experiments are performed: (1) Three pairs of IR images and RGB images as input respectively, we compare the effects of SR with single image input and two image input; (2) SR performance comparisons with state-of-the-arts algorithm by the upsampling scale of 2 and 4. we introduce the datasets for training and testing as follows.

**Figure 8:**
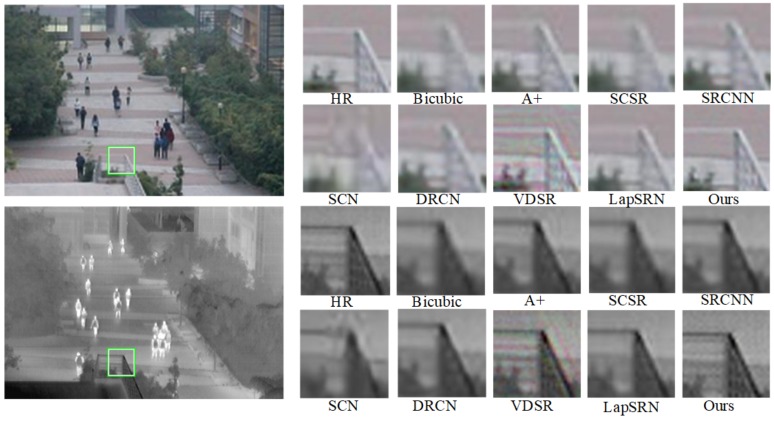
SR experiment results of scale 2 in Teaching building.

**Figure 9:**
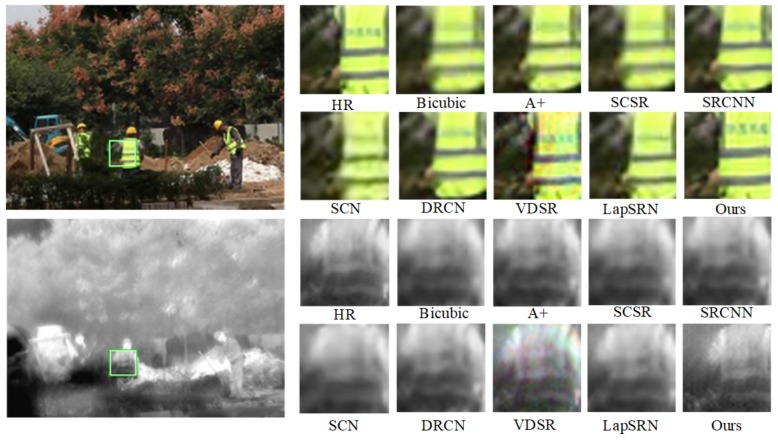
SR experiment results of scale 2 in Laborer.

**Figure 10:**
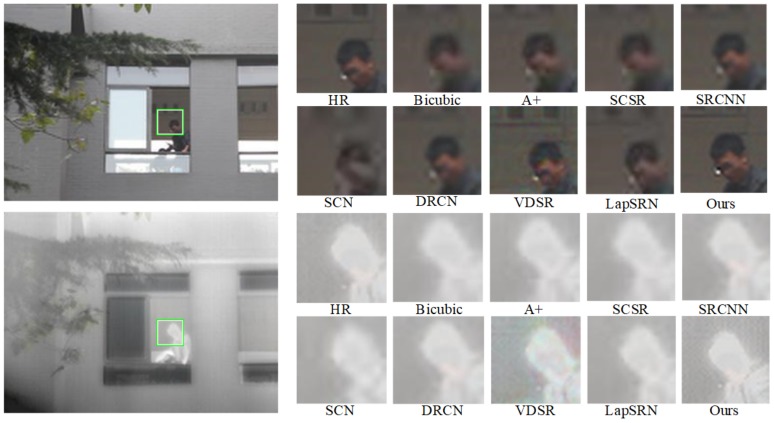
SR experiment results of scale 2 in Window.

Qualitative and quantitative performance of our model in comparison with state-of-the-art ones: A+, SCSR, SCN [35], SRCNN, VDSR, LapSRN, are presented as follows to evaluate the results. Peak Signal Noise Rate (PSNR) and Self-Similarity (SSIM) are used as the quality metric to evaluate the performance of all methods. Besides, visual comparisons on IR-COLOR2000 dataset are shown in Figure 8, Figure 9 and Figure 10 with the scale factor of 2. SR quantitative results of scale ×2 and ×4 are given in Table 3. In Figure 8, regions highlighted by green rectangles are magnified, and the difference between SR image and ground truth is clear for easy visual inspection. The result of RGB-IR cross input and sub-pixel upsampling network performance well in image reconstruction and simulation run at high speed.

We use PSNR and SSIM to evaluate the performance of our datasets. SSIM metric indicated the pixel similarity and local structure similarity between reconstructed HR image and ground truth. In Table 3, the performance of the proposed method is higher than A+, SCSR, SCN, SRCNN, VDSR, DRCN, LapSRN, especially on a scale of 4. Figure 8, Figure 9 and Figure 10 show SR experiment results by different algorithms. Reconstructed contextual information of the proposed method including edges and textures more clear than the others such as ailing in the Building, the waistcoat of Laborer, and the head of the man in Figure 8, Figure 9 and Figure 10.

### 4.2. Comparison to the State-of-the-Art

Since the previous image super-resolution reconstruction is pure infrared image super-resolution or visible image super-resolution. Furthermore, the existing input multi-modal data is all for image fusion, no dual input super-resolution reconstruction algorithm for images. For the sake of fairness, this paper makes more comparisons with single-input super-resolution reconstruction algorithms. Different from image fusion, this paper uses infrared and visible images for image super-resolution. Pairs of IR images and RGB images as input respectively, we compare the effects of SR with single image input and two images. To compensate for the contrast algorithm without double input, I will increase more single input super-resolution contrast experiments. Three pairs of images from the KAIST [36] and OutdoorUrban [37] dataset are used to test the performance of the proposed algorithm under low illumination.

**Figure 11:**
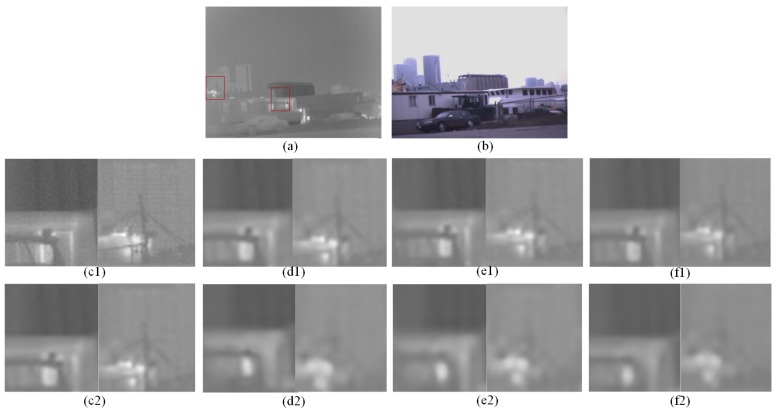
The visual quality comparison for an image from OutdoorUrban datasets with scale ×2 and ×4.(**a**) is infrared image as input (**b**) is the RGB image as input. (**c1**–**f1**) is SR results for ×2 scale respectively proposed method, vdsr, srcnn, A+. (**c2**–**f2**) is SR results for ×4 scale respectively proposed method, vdsr, srcnn, A+.

**Figure 12:**
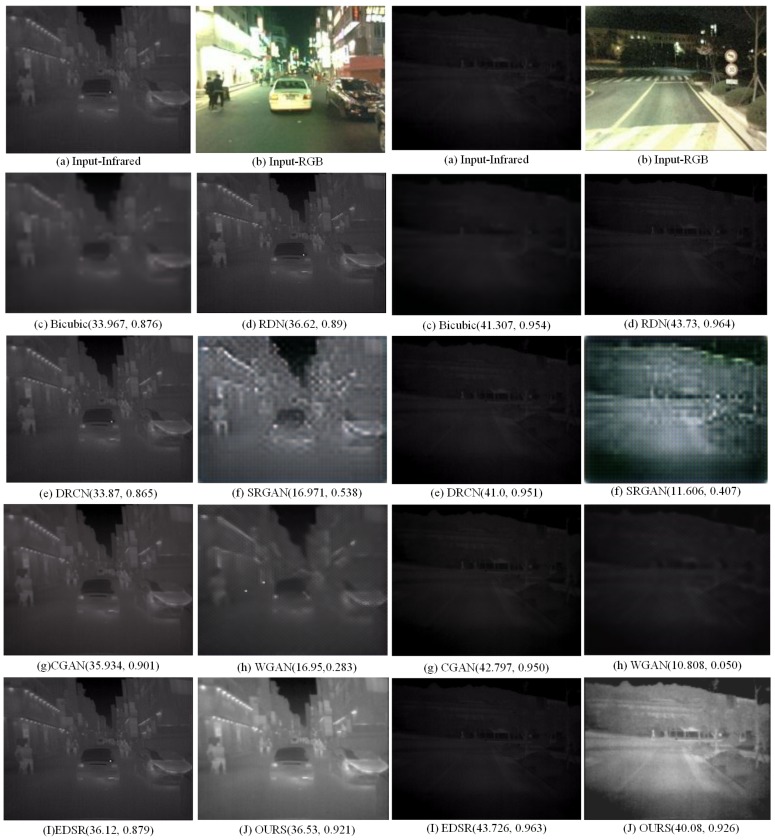
SR experiment results of scale 4 in night from KAIST dataset.

To further highlight the performance of our SR method, a set of images in low light were selected for simulation experiments in Figure 11. OutdoorUrban dataset is images fusion datasets made by Nigel J. W. Morri in the *Statistics of Infrared Images* [37]. The contrast of the 4× scales experiment is performed by a set of dimly light images using the algorithms Bicubic, DRCN [10], RDN [38], SRGAN [39], CGAN [40] and WGAN [41] and proposed method which embedded the visible features into the infrared image in Figure 12. The PSNR and SSIM of Figure 11 and Figure 12 are shown in Table 4.

The experiment on night images from the KAIST dataset is shown in Figure 12. Clear RGB images is helpfuly for infrared images recovery, and improved SR results can be seen in Figure 12h. Also, the reconstruction image makes the inconspicuous features of infrared image clearer, which is consistent with human visual features. However, when the RGB Image is not clear enough, our method hasn’t any advantages of the image SR. It is obvious that the partial brightness of the lamp in the figure is different when the super-resolution scale is four times and two times, and the effect of the proposed method is indeed better than the others especially the evaluation values in Figure 12.

### 4.3. Analysis& Discussion

**Figure 13:**
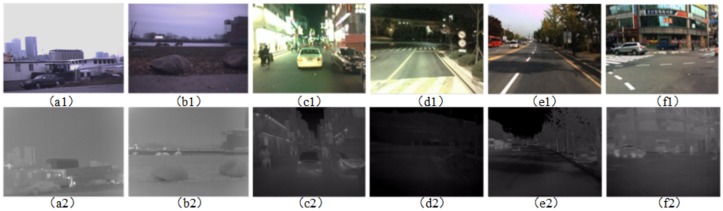
Test images in Table 4 ((**a**,**b**) are from OutdoorUrban dataset; (**c**,**d**) are from night videos of KAIST dataset; (**e**,**f**) are from daylight videos of KAIST dataset.).

Six pairs of images from KAIST [36] and OutdoorUrban [37] dataset respectively as Figure 13 are chosen for SR experiments on A+, SCSR, SRCNN, DRCN, SRGAN, WGAN, CGAN, and our method. Infrared images and visible images are from two datasets of OutdoorUrban and KAIST. Figure 13a,b are in slightly weak light; Figure 13c,d are in low light; Figure 13e,f are in normal light. The PSNR and SSIM of Figure 13 are in Table 4.

Further verifying the stability of the algorithm, the data in KAIST and OutdoorUrban are chosen for quality comparison in Table 4.

The CGAN, WGAN, SRGAN, and RDN in Table 4 are not models for infrared images, so the experimental results are not optimal. The data in Table 4 shows that the algorithm in this paper is more stable than RDN and GAN-based methods under weak light and normal light conditions. The Evaluation indexes of Table 4 marked in blue are the best results of image SR. When visible light is low, the algorithm in this paper loses some advantages. In general, it can be seen from the mean signal-to-noise ratio of the six groups of experiments that the proposed algorithm is more stable than other algorithms.

**Figure 14:**
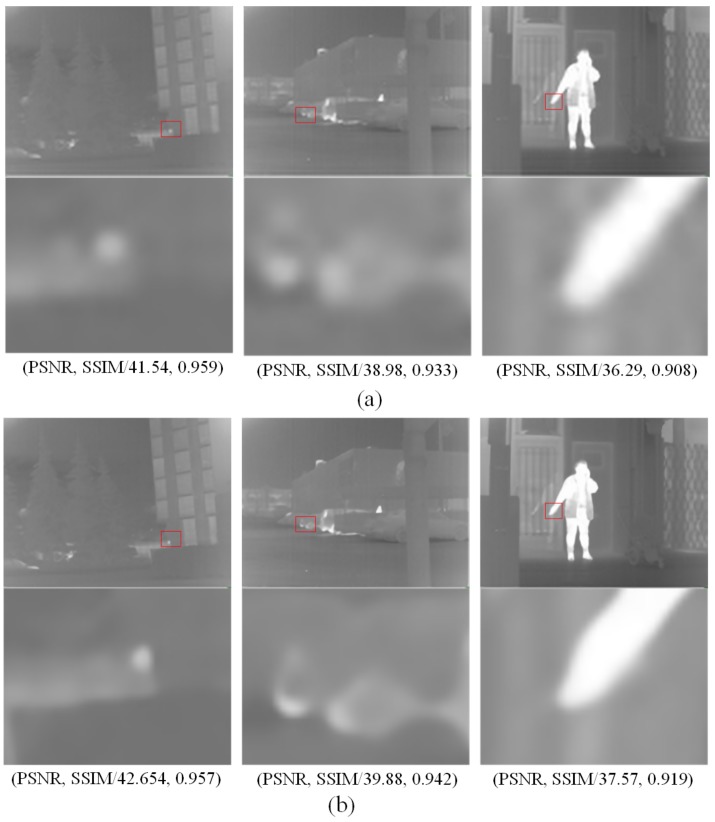
Comparison on the proposed method without denoising and the proposed method with denoising. (**a**) is the result of the proposed method without denoising; (**b**) is the result of the proposed method with denoising.

To prove that infrared image noise reduction is effective for super-resolution networks, we conducted an experiment on three pairs of images from the OutdoorUrban [37] dataset. We use the proposed algorithm to reconstruct the above three pairs of images as shown in Figure 14 and calculate the image quality evaluation results. The results without denoising process are shown in Figure 14a and the results with denoising are shown in Figure 14b.

As can be seen in Figure 14, the results of group (b) are better than that of group (a). For the evaluation values of PSNR and SSIM, it can be noticed that the PSNR of (b) is nearly 1dB higher than (a). In addition, the image similarity parameters SSIM has not decreased significantly. This indicates that our denoising scheme can improve performance.

Since RGB-IR cross input and sub-pixel upsampling network make full use of the advantage of multiple model images, the network obtained a larger receptive field and less noise infrared image. Experimental results show that the proposed algorithm is very successful and effectively to improve image resolution.

### 4.4. Running Time

In this work, we can design and train different pre-trained models. Also, we need not train the model with odd scales, because the proposed network could handle the upsampling rate is odd. When dealing with an odd scale such as 3×, the input image is upsampled to the nearest scale by 4×, then the super-resolution result is downsampled to the target scale. Moreover, our method is less time consumption in comparison to the methods A+, SCN, SRCNN, VDSR, DRCN, RDN [38], SRGAN [39], CGAN [40] and WGAN [41] as illustrated in Table 5.

The running time is related to the depth and the input size of the network. The structure of SCN, SRCNN, and VDSR are relatively simple, but the input image size is larger than our proposed network because the input of that three networks are directly up-sampled to the ideal size, but our algorithm is gradually up-sampled to the ideal size. Thus the inference time of our proposed is shorter than these networks. The structure of DRCN, RDN, SRGAN, CGAN, and WGAN algorithms are deeper than our algorithm, which corresponds to longer inference time.

## 5. Conclusions

In this paper, we presented two input networks that employ the guided upsampling Laplacian Pyramid Network for super-resolution. The deep network can be optimized to be faster via guided filtering and sub-pixel convolution upsampling step by step. A significant improvement can be shown visually by using an RGB image to guide the IR input image and combining the RGB feature image with the IR feature image. The proposed RGB-IR cross input and sub-pixel upsampling network reduced the IR image noise problem and improved the IR super-resolution image quality by adding RGB details. Relative evaluations on datasets demonstrate that the proposed algorithm performs satisfactorily compared to the other SR methods in terms of visual quality. 

## Figures and Tables

**Table 1:** Comparison of CNN-based SR methods: SRCNN, FSRCNN, VDSR, DRCN, and LapSRN.

Method	Input	Depth	Residual Structure	Reconstruction	Loss
SRCNN	LR + bicubic	3	×	-	L2
FSRCNN	LR	8	×	-	L2
VDSR	LR + bicubic	20	*√*	-	L2
DRCN	LR + bicubic	5 (recursive)	×	-	L2
LapSRN	LR	24	*√*	Progressive	Charbonnier

**Table 2:** Convolution structure of network branches for 4× SR.

Part	Convolution Kernel Size		Depth	Filters	Parameters	Input Dimensions	Output Dimensions
Feature Extraction	3×3		22	64	737k	1	64
	sub-pixel	3×3	2	64	36k	64	32
		2×2	2	32	16k	32	64
Image Reconstruction	4×4		2	64	1024	1	1

**Table 3:** Quantitative evaluation of state-of-art SR algorithm.

Algorithm	Scale	Building		Laborer		Window	
		RGB	IR	RGB	IR	RGB	IR
		PSNR/SSIM	PSNR/SSIM	PSNR/SSIM	PSNR/ SSIM	PSNR/SSIM	PSNR/SSIM
Bicubic	×2	32.858/0.901	34.755/0.908	28.202/0.826	37.436/0.933	32.137/0.909	40.118/0.938
A+		32.913/0.912	35.01/0.921	29.011/0.921	37.867/0.940	33.973/0.929	40.563/0.941
SCSR		33.158/0.906	35.055/0.913	28.502/0.837	37.736/0.934	32.431/0.928	40.417/0.939
SCN		31.484/0.715	33.468/0.537	27.204/0.730	35.078/0.653	32.312/0.555	38.159/0.506
SRCNN		35.051/0.936	36.144/0.924	30.019/0.884	38.459/0.945	34.888/0.933	40.777/0.945
VDSR		34.420/0.922	35.802/0.913	29.573/0.871	38.200/0.936	34.078/0.923	40.609/0.939
LapSRN		34.440/0.929	35.815/0.920	29.580/0.876	38.207/0.943	34.083/0.930	40.616/0.945
Ours		**34.915/0.945**	**36.190/0.929**	**30.171/0.880**	**38.836/0.948**	**34.682/0.943**	**41.150/0.949**
Bicubic	×4	26.042/0.627	27.971/0.748	23.783/0.529	31.886/0.8213	27.747/0.767	35.224/0.867
A+		28.912/0.712	30.213/0.810	24.871/0.592	33.248/0.849	29.436/0.818	36.989/0.899
SCSR		26.339/0.649	38.311/0.768	23.857/0.528	32.221/0.826	27.902/0.902	35.550/0.876
SCN		26.151/0.636	28.129/0.759	24.537/0.569	32.408/0.839	28.962/0.790	35.727/0.889
SRCNN		29.365/0.767	31.445/0.834	25.164/0.640	34.310/0.876	30.292/0.844	37.535/0.904
VDSR		29.382/0.754	31.101/0.813	25.129/0.638	34.122/0.860	30.253/0.835	37.070/0.891
LapSRN		29.392/0.766	31.110/0.824	25.138/0.641	34.133/0.871	30.264/0.844	37.079/0.898
Ours		**29.989/0.782**	**31.890/0.850**	**25.82/0.650**	**34.697/0.885**	**30.762/0.864**	**37.805/0.910**

**Table 4:** Quantitative evaluation of a few images from KAIST and OutdoorUrban dataset.

Image	Scale	Index	Bicubic	A+	SRCNN	SCSR	CGAN	WGAN	DRCN	RDN	SRGAN	Proposed
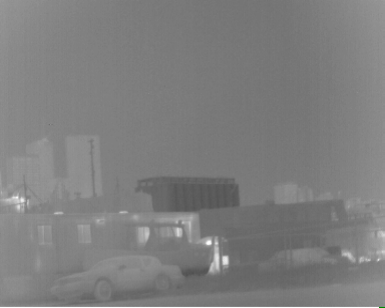	×2	PSNR	42.400	43.4	43.635	42.4244	-	17.896	44.17	44.21	24.538	44.704
		SSIM	0.9592	0.963	0.965	0.9603	-	0.364	0.976	0.977	0.503	0.987
	×4	PSNR	38.955	36.093	39.723	39.2457	39.983	14.78	39.98	40.47	20.89	40.31
		SSIM	0.938	0.887	0.944	0.9385	0.950	0.587	0.942	0.949	0.581	0.958
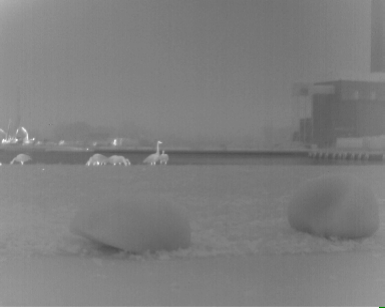	×2	PSNR	42.614	43.2	43.303	42.369	-	24.924	44.101	44.43	24.589	45.087
		SSIM	0.964	0.971	0.967	0.962	-	0.396	0.976	0.979	0.595	0.986
	×4	PSNR	39.124	38.3	39.855	39.313	39.975	17.874	39.59	40.54	20.456	40.06
		SSIM	0.9377	0.94	0.944	0.9377	0.948	0.587	0.949	0.945	0.526	0.960
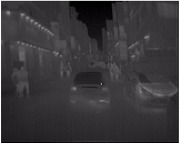	×2	PSNR	38.946	39.9	40.432	39.01	-	18.313	38.392	41.21	29.207	41.929
		SSIM	0.946	0.953	0.959	0.948	-	0.205	0.941	0.970	0.655	0.972
	×4	PSNR	33.967	34.6	35.762	34,3	35.934	16.95	33.87	36.52	16.971	36.53
		SSIM	0.876	0.876	0.889	0.872	0.901	0.283	0.865	0.89	0.5386	0.921
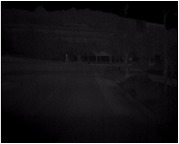	×2	PSNR	46.618	46.5	46.253	46.4	-	13.045	43.891	47.36	34.96	45.50
		SSIM	0.975	0.974	0.978	0.968	-	0.053	0.967	0.967	0.806	0.916
	×4	PSNR	41.307	41.9	42.942	41.6	42.797	10.808	41.0	43.73	11.616	40.48
		SSIM	0.954	0.952	0.957	0.953	0.950	0.050	0.951	0.964	0.407	0.920
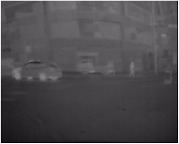	×2	PSNR	54.188	54.4	54.82	53.9	-	15.203	54.74	54.31	27.469	56.837
		SSIM	0.976	0.981	0.986	0.976	-	0.120	0.982	0.981	0.638	0.991
	×4	PSNR	46.1	46.26	46.305	46.3	41.283	13.738	44.74	44.53	12.752	47.196
		SSIM	0.978	0.980	0.982	0.981	0.968	0.244	0.976	o.980	0.582	0.992
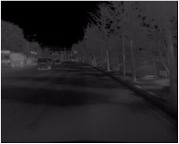	×2	PSNR	53.5	54.0	54.732	54.6	-	17.393	54.979	54.23	32.82	57.42
		SSIM	0.956	0.964	0.968	0.960	-	0.157	0.971	0.969	0.741	0.984
	×4	PSNR	44.275	46.08	47.26	46.3	43.062	15.686	44.979	45.12	16.397	47.71
		SSIM	0.915	0.924	0.968	0.924	0.958	0.205	0.949	0.96	0.582	0.979
**Average**	×2	PSNR	46.379	46.9	47.197	46.450	-	17.795	46.712	47.625	28.930	48.579
	×4	PSNR	40.621	40.538	41.974	41.176	40.505	14.972	40.693	41.835	16.513	42.047

**Table 5:** Comparison of running time for 4× in our datasets.

Methods	A+	SCN	SRCNN	VDSR	DRCN	EDSR	RDN	SRGAN	CGAN	WGAN	Ours
**Running time/ Sec**	2.163	5.340	0.728	0.246	8.215	0.612	0.469	0.293	0.288	0.150	0.098

## References

[1] Chang H., Yeung D.Y., Xiong Y. Super-resolution through neighbor embedding. Proceedings of the 2004 IEEE Computer Society Conference on Computer Vision and Pattern Recognition.

[2] Timofte R., De V., Gool L.V. Anchored Neighborhood Regression for Fast Example-Based Super-Resolution. Proceedings of the IEEE International Conference on Computer Vision (ICCV).

[3] Timofte R., Smet V.D., Gool L.V. A+: Adjusted Anchored Neighborhood Regression for Fast Super-Resolution. Proceedings of the Asian Conference on Computer Vision.

[4] Jianchao Y., John W., Thomas H., Yi M. (2010). Image Super-Resolution via Sparse Representation. IEEE Trans. Image Process..

[5] Zhao Y., Chen Q., Sui X., Gu G. (2015). A novel infrared image super-resolution method based on sparse representation. Infrared Phys. Technol..

[6] Qi G., Wang H., Haner M., Weng C., Chen S., Zhu Z. (2019). Convolutional neural network based detection and judgement of environmental obstacle in vehicle operation. CAAI Trans. Intell. Technol..

[7] Dong C., Loy C.C., He K., Tang X. (2015). Image SuperResolution Using Deep Convolutional Networks. IEEE Trans. Pattern Anal. Mach. Intell..

[8] Dong C., Chen C.L., Tang X. Accelerating the Super-Resolution Convolutional Neural Network. Proceedings of the European Conference on Computer Vision.

[9] Kim J., Lee J.K., Lee K.M. Accurate Image Super-Resolution Using Very Deep Convolutional Networks. Proceedings of the IEEE Conference on Computer Vision and Pattern Recognition.

[10] Kim J., Kwon Lee J., Mu Lee K. Deeply-recursive convolutional network for image super-resolution. Proceedings of the IEEE Conference on Computer Vision and Pattern Recognition.

[11] Ma J., Wang X., Jiang J. (2019). Image Super-Resolution via Dense Discriminative Network. IEEE Trans. Ind. Electron..

[12] Ma J., Yu W., Liang P., Li C., Jiang J. (2019). FusionGAN: A generative adversarial network for infrared and visible image fusion. Inf. Fusion.

[13] Han T.Y., Kim Y.J., Song B.C. Convolutional neural network-based infrared image super resolution under low light environment. Proceedings of the 25th European Signal Processing Conference (EUSIPCO).

[14] Tseng C.W., Su H.R., Lai S.H., Liu J. Depth image super-resolution via multi-frame registration and deep learning. Proceedings of the 2016 Asia-Pacific Signal and Information Processing Association Annual Summit and Conference (APSIPA).

[15] Huang D., Huang W., Gu P., Liu P., Luo Y. (2017). Image super-resolution reconstruction based on regularization technique and guided filter. Infrared Phys. Technol..

[16] Lai W.S., Huang J.B., Ahuja N., Yang M.H. (2018). Fast and accurate image super-resolution with deep laplacian pyramid networks. IEEE Trans. Pattern Anal. Mach. Intell..

[17] Russakovsky O., Deng J., Su H., Krause J., Satheesh S., Ma S., Huang Z., Karpathy A., Khosla A., Bernstein M. (2015). ImageNet Large Scale Visual Recognition Challenge. Int. J. Comput. Vision.

[18] Huang J.B., Singh A., Ahuja N. Single Image Super-resolution from Transformed Self-Exemplars. Proceedings of the IEEE Conference on Computer Vision and Pattern Recognition.

[19] Fujimoto A., Ogawa T., Yamamoto K., Matsui Y., Aizawa K. Manga109 dataset and creation of metadata. Proceedings of the 1st International Workshop on coMics ANalysis, Processing and Understanding.

[20] Martin D.R., Fowlkes C., Tal D., Malik J. A Database of Human Segmented Natural Images and its Application to evaluating segmentation algorithms and measuring ecological statistics. Proceedings of the Eighth International Conference On Computer Vision (ICCV-01).

[21] Choi Y., Kim N., Hwang S., Kweon I.S. Thermal Image Enhancement using Convolutional Neural Network. Proceedings of the 2016 IEEE/RSJ International Conference on Intelligent Robots and Systems (IROS).

[22] Barrera F., Lumbreras S.A.D. (2013). Multispectral piecewise planar stereo using Manhattan-world assumption. Pattern Recognit. Lett..

[23] Hwang S., Park J., Kim N., Choi Y., Kweon I.S. Multispectral Pedestrian Detection: Benchmark Dataset and Baseline. Proceedings of the IEEE Conference on Computer Vision and Pattern Recognition.

[24] Zou X. Research on Infrared and Visible Image Registration Algorithm Based on Self-similarity. Proceedings of the International Conference on Information Science and Technology (IST 2017).

[25] Rudin L.I., Osher S., Fatemi E. (1992). Nonlinear total variation based noise removal algorithms. Physica D.

[26] Zhang X., Wang R., Jiao L.C. Partial Differential Equation Model Method Based on Image Feature for Denoising. Proceedings of the 2011 International Workshop on Multi-Platform/Multi-Sensor Remote Sensing and Mapping.

[27] Pu Y.F., Siarry P., Zhou J.L., Liu Y.G., Zhang N., Huang G., Liu Y.Z. (2014). Fractional partial differential equation denoising models for texture image. Sci. Chin. Inf. Sci..

[28] Dabov K., Foi A., Egiazarian K. Image denoising with block-matching and 3D filtering. Proceedings of the Image Processing: Algorithms and Systems, Neural Networks, and Machine Learning.

[29] Honzátko D., Kruliš M. (2017). Accelerating block-matching and 3D filtering method for image denoising on GPUs. J. Real-Time Image Process..

[30] Guan J., Lai R., Xiong A. (2019). Wavelet Deep Neural Network for Stripe Noise Removal. IEEE Access.

[31] Tan W., Zhou H., Rong S., Qian K., Yu Y. (2018). Fusion of multi-focus images via a Gaussian curvature filter and synthetic focusing degree criterion. Appl. Opt..

[32] He K., Sun J., Tang X. Guided image filtering. Proceedings of the European Conference on Computer Vision.

[33] Wu H., Shuai Z., Zhang J., Huang K. Fast End-to-End Trainable Guided Filter. Proceedings of the IEEE Conference on Computer Vision and Pattern Recognition.

[34] Shi W., Caballero J., Theis L., Huszar F., Wang Z. (2016). Is the deconvolution layer the same as a convolutional layer?. arXiv.

[35] Wang Z., Liu D., Yang J., Han W., Huang T. Deep networks for image super-resolution with sparse prior. Proceedings of the IEEE International Conference on Computer Vision.

[36] Lee S., Cho M.S., Jung K., Kim J.H. Scene text extraction with edge constraint and text collinearity. Proceedings of the 2010 20th International Conference on Pattern Recognition.

[37] Morris N.J.W., Avidan S., Matusik W., Pfister H. Statistics of Infrared Images. Proceedings of the 2007 IEEE Conference on Computer Vision and Pattern Recognition.

[38] Zhang Y., Tian Y., Kong Y., Zhong B., Fu Y. Residual Dense Network for Image Super-Resolution. Proceedings of the IEEE Conference on Computer Vision and Pattern Recognition.

[39] Ledig C., Theis L., Huszár F., Caballero J., Cunningham A., Acosta A., Aitken A., Tejani A., Totz J., Wang Z. Photo-realistic single image super-resolution using a generative adversarial network. Proceedings of the IEEE conference on computer vision and pattern recognition.

[40] Yuyang W., Feng S., Ye Q. Super-Resolution of Text Image Based on Conditional Generative Adversarial Network. Proceedings of the Pacific Rim Conference on Multimedia.

[41] Yu L., Long X., Tong C. Single Image Super-Resolution Based on Improved WGAN. Proceedings of the 2018 International Conference on Advanced Control, Automation and Artificial Intelligence (ACAAI 2018).

